# Implementation of a Novel Concept of Emergency Department Management: e-Boss

**DOI:** 10.3390/ijerph191912291

**Published:** 2022-09-27

**Authors:** Thomas Schmutz, Khaled Habchi, Christophe Le Terrier, Catherine Favre Kruit, Patricia Stengel, Youcef Guechi, Vincent Ribordy

**Affiliations:** 1 Department of Emergency Medicine, Fribourg Hospital, The University of Fribourg, 1708 Fribourg, Switzerland; 2 Division of Intensive Care, Department of Acute Medicine, Geneva University Hospitals and University of Geneva, 1205 Geneva, Switzerland; 3 Communication Department, Fribourg Hospital, 1708 Fribourg, Switzerland

**Keywords:** emergency, social media, management, emergency department

## Abstract

Hospital-based emergency services are frequently criticized worldwide for their management, which can lead to a decrease in staff motivation, with a potential impact on patient safety. This article describes how harnessing the power of social networks can facilitate the management of emergency department teams. Beyond teaching, promoting emergency medicine and recruiting health professionals, these tools can unite employees around a virtual leader and help develop a true service culture. The concept of management through social networks is a novel manner to reach out to staff and should be further explored for use in the health care context.

## 1. Introduction

Since the end of the 2000s, social networks have profoundly changed our behavior. By creating Facebook (https://facebook.com (accessed on on 1 August 2022)) in 2004, Mark Zuckerberg is at the origin of this craze. Facebook Meta now has almost 3 billion active monthly users with an ever-growing number of followers. A new lexical field has even opened up, e.g., we “post”, “tweet”, “influence”, and “blacklist”. “Scrolling”, a characteristic gesture of the new digital era consisting of scrolling through content on a screen, is a testimony to the overabundance of digital information at our disposal. Besides being addictive for some people, this digital behavior is the symbol of a new generation. Widely used by the population, social networks have now become a preferred communication channel for many personalities or institutions, whether public or private. Most hospitals have understood this, and many already have an official account on one or more of these networks. Social media platforms are now great information distribution channels, and the medical sector is no exception [1]. The literature shows a wide use of social networks in the health field including for health promotion, career development or practice promotion, recruitment, professional networking and destressing, professional medical education, telemedicine, scientific research, and critical public health care issues [2,3]. The fields of application appear limitless, and everything seems possible. Nevertheless, in the hospital culture, caution has always been required [4].

However, the COVID-19 crisis has shaken up the established rules and the pandemic has acted as a catalyst for social networks. Social media have been highly criticized for their misinformative nature (fake news) and the management of the pandemic has been greatly impacted by the influence of this phenomenon [5]. The World Health Organization and other institutions have used the concept of an “infodemic” to denounce “deliberate attempts to disseminate misinformation aimed at undermining public health actions and promoting the alternative objectives of certain groups or individuals”. In this context, many medical teams felt the need to speak directly to the population and several critical care services (emergency, intensive care) have posted their daily lives to show their reality.

Following this trend, the head of the emergency unit of the cantonal hospital of Fribourg in Switzerland decided, in the middle of the COVID-19 pandemic, to open an account on the social media platform Instagram. This account was, and remains, separate from the official hospital account. [6]. This social media account allowed him to share the daily life of his team. Two years later the importance of this account has grown, exceeding its initial objectives, and transformed into a management tool. Few teams describe this use in the health sector. It is therefore important to share this concept, based on our experience, because it could be useful to other managers. This article uses a narrative approach to describe the birth of this concept, its initial objectives, and the risks and obstacles to its development. Finally, we address the impact of this new tool in the management of an emergency service.

## 2. Risks Related to the Use of Social Networks in Hospitals

From the first publications on Instagram, the hospital’s communication service took notice, especially to the account’s humorous tone. It alerted the general management of the institution and asked for the account to be closed, insisting on the risks and the shortcomings of such an initiative promoted by staff who are not professionals in the field of communication. Indeed, posting a comment, a photo or even a video is not a trivial matter and it is not always easy to communicate from a medical point of view while securing the data. Before any publication, it is essential to obtain the patient’s consent, even if they can only be recognized in a very indirect way (dates, times, context).

The disclosure of medical information without this agreement on a social platform is punishable by a severe conviction. The penal and ethical consequences are therefore substantial and the “faux pas” of a professional and, by extension, of their institution, will be immediately sanctioned by public opinion. The risk of violation is permanent; an error or an oversight can be irreversible in the event of untimely publication and the “viral” nature of the content published. Each “post” can be sent to an infinite number of people, sometimes with a totally erroneous interpretation of the situation in the absence of contextualization. Several recommendations for the use of social media have emerged to protect physicians’ personal information and privacy [7,8,9]. In the field of emergency medicine, guidance for good practice has also been published, notably by policy statements of the American College of Physicians [10].

Hospitals rely on the work of a communication unit dedicated to managing the hospital’s image. The primary objective of this unit is to provide information that promotes the diversity and quality of the services provided to users, while taking care to avoid generating controversy or misunderstandings that would damage its image. Health professionals can sometimes find it difficult to identify with this image, which is sometimes considered too polished or out of touch with their daily lives. These employees may decide to communicate using their own names, but this exposure carries risks, and the release of inappropriate content can have adverse consequences for the institution and, ultimately, the employee. Indeed, by posting inappropriate content on the web, the caregiver can trigger a “digital chain reaction” that can quickly take an unfortunate negative turn [11,12].

## 3. Origin of the Concept

**Figure 1:**
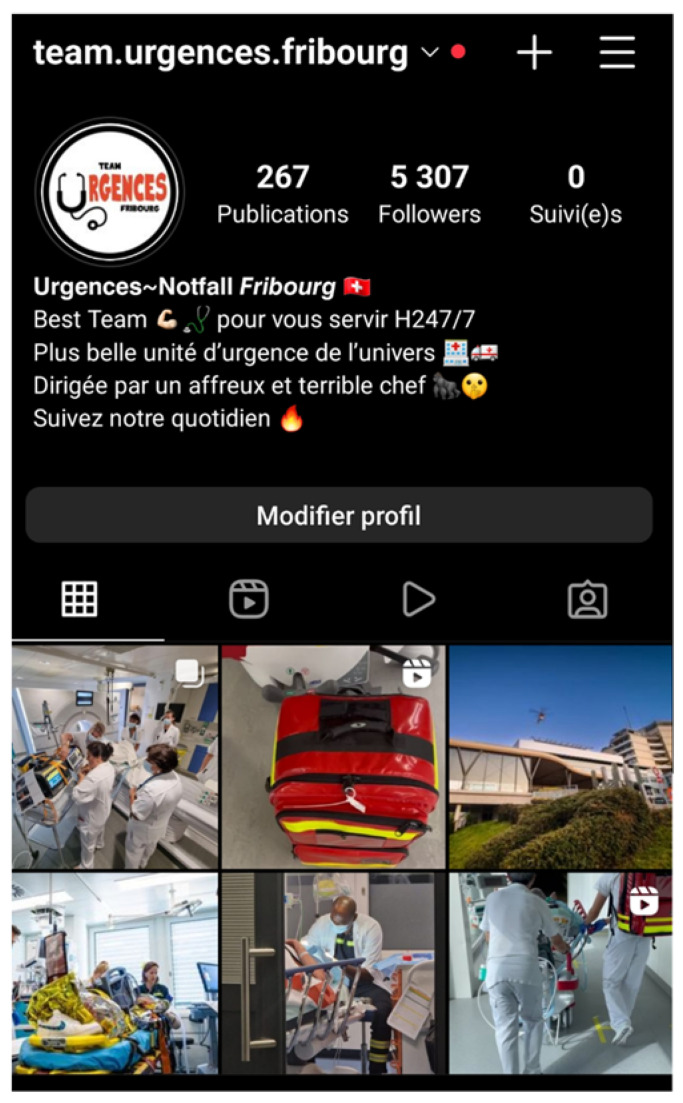
Smartphone screenshot of the Fribourg hospital emergency department Instagram account main menu. The account profile reads: Follow the daily life of the best team of the most beautiful emergency unit in the universe, led by an ugly and terrible boss.

The purpose of the account (Figure 1) was to boost the service’s image, which was losing momentum, and to give present a more human and appealing dimension to their work. For this, Instagram seemed to be the best choice: Instagram is a social network that has the reputation of being rather caring and kind, with external reactions to content more easily avoided or controlled, unlike Twitter or Facebook, where the slightest slip-up leads to a cascade of chain reactions, sometimes of a very aggressive nature [11,12]. The network was founded in 2010 and allows the sharing of photos and videos. Since 2012, the application has belonged to the American group Meta (formerly Facebook Inc.) and claims to more than 1 billion users worldwide. It allows the rapid distribution of photographic and video content to users interested in specific keywords (hashtags). For example, the French language hashtag #urgences has 75,000 publications (1.9 million for the English equivalent, #emergency). Users can then subscribe to accounts (created by other users) corresponding to their interests. Exchanges in this network are particularly active; everyone can illustrate their daily life with original content (high quality photography, video production, informative text, etc.). The Instagram network allows for the distribution of simple, generally photographic content that is accessible at a glance. Many physicians have already understood this and are sharing content on this network.

**Figure 2:**
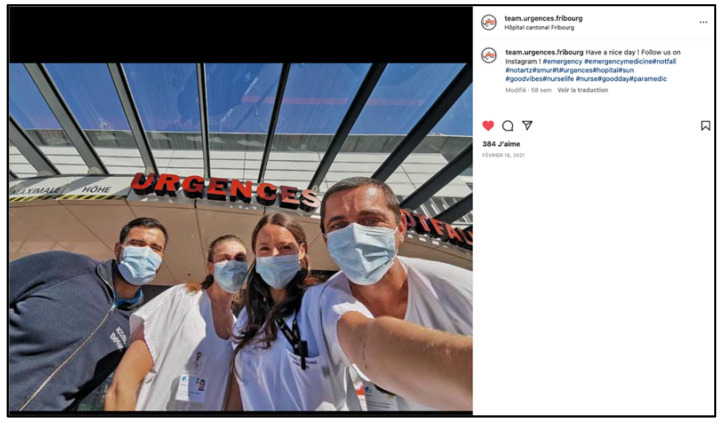
Screenshot of an Instagram “post” illustrating good team cohesion.

The boss therefore began to highlight the work of their employees with patients by day and by night, during the week, and at weekends. Posting takes place at any time of the day or night in a rather spontaneous way, the main objective being to highlight the work accomplished by staff and to increase the attractiveness of the service (Figure 2). As it is not the hospital’s institutional communication channel, the tone used on the account can afford to take a deliberately offbeat, playful, humorous approach that goes against the very codes of a hospital institution where rigor and seriousness are required.

## 4. Initial Objectives

### 4.1. Recruitment

Similar to most emergency services in Switzerland, the one in Fribourg is regularly exposed to medical and nursing recruitment difficulties. In 2020, the situation became critical. Despite the publication of classic recruitment advertisements, the service struggled to build a team due to a lack of visibility. The shortage of emergency physicians in Europe (abandonment of the profession due to professional exhaustion) and in Switzerland (non-recognition of a specialist title), as well as the increasing number of overstretched physicians who have to deal with staff shortages, has further aggravated the issue, leading to departures [13]. The non-border nature of the canton of Fribourg, which imposes the expatriation of foreign physicians, and its bilingualism (French and German) also limit applications.

**Figure 3:**
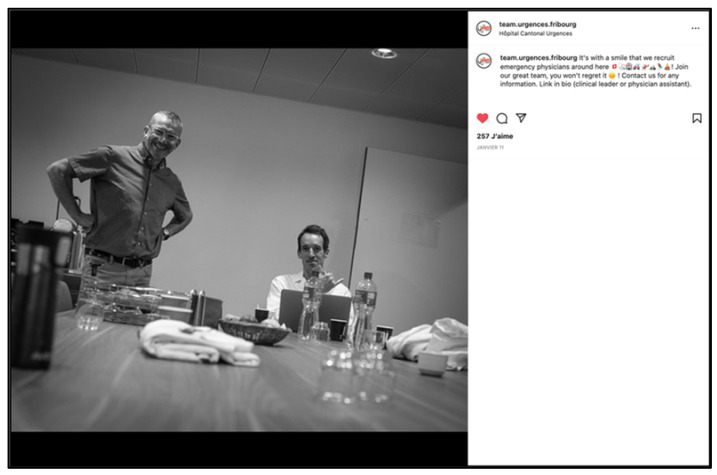
Images of the management team highlighting humanization and personalization of the recruitment process (screenshot).

**Figure 4:**
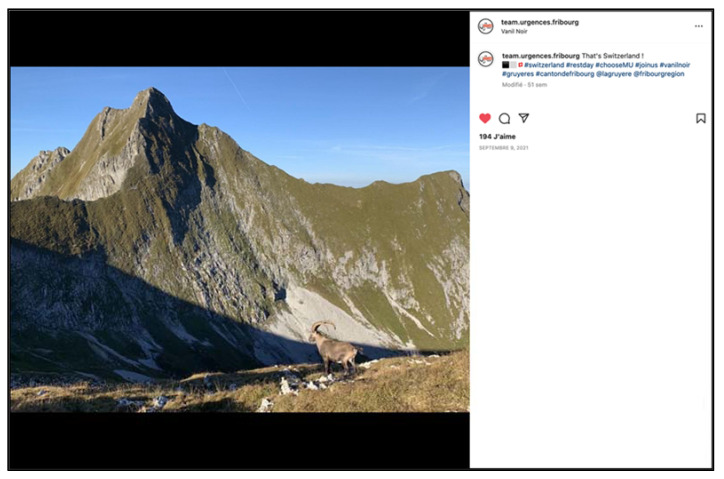
Highlighting the living environment which plays a major role in the recruitment of new employees: the Fribourg region in Switzerland offers many opportunities for extra-professional activities (screenshot).

In addition to outlining the measures already put in place (caring management, day and night rest periods for employees, renovation of premises, training, team dynamism) to enhance attractiveness and improve patient care, the primary objective of creating this account was to provide a direct showcase of the service. The regular promotion of the management team’s objectives (quality of life at work, #lovemyjob), as well as the pleasant living conditions in the canton of Fribourg (#afterwork), arouse the curiosity of healthcare professionals (Figure 3 and Figure 4). When a new employee makes contact or is hired, they are encouraged to follow the account so that they have a sense of familiarity with the team, the department and its organization when they start.

### 4.2. Promoting Emergency Medicine

In Europe, since the advent of the television series ER (Emergency Room) in 1994 in the USA, the number of programs, TV movies and films dealing with the daily life of the emergency room has grown. The directors well understood that every day in the emergency room is different (activity, severity of patients, type of pathologies) and that the possibilities of communication on this theme are many and varied, with the emergency room being at the crossroads of the general population and the hospital and its specialties. With a little skill, it is quite easy to quickly unite a community of non-healthcare followers on this topic and give credibility to a social media account focused on this topic, much more so than other medical specialties focused on one type of practice.

**Figure 5:**
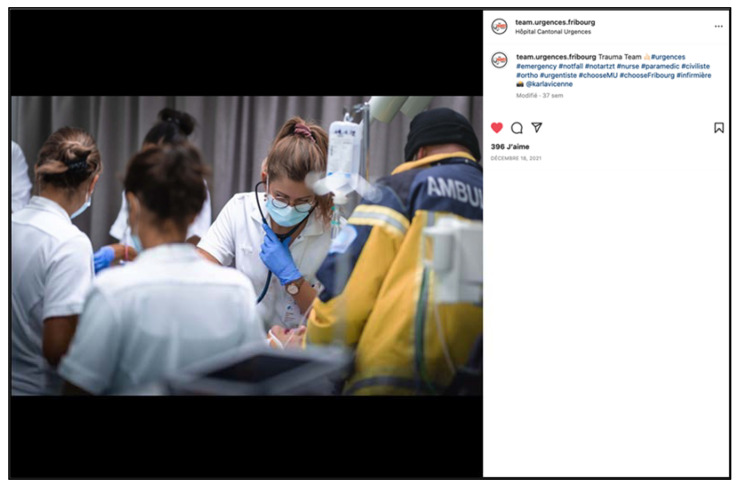
Promoting the work of teams at Fribourg hospital (screenshot).

In Switzerland, emergency medicine is still not recognized as a specialty as such, contrary to most countries of the European Union [14]. This lack of recognition is detrimental to vocations, as physicians-in-training prefer to turn to a more highly valued specialist title. The arduousness of emergency room work (staggered hours, night and weekend work, etc.), amplified year after year by the ever-increasing influx of patients, reinforces the rejection of the profession. Emergency departments suffer because chronic understaffing is closely linked to the hardship experienced by the teams. However, emergency medicine is a rich specialty. Its development in recent years has been exponential (pre-hospital emergency medicine, research and publications, clinical ultrasound, etc.) and the possibilities of promoting this specialty through social networks are infinite (Figure 5). Indeed, some publications in the form of reels gain up to 120,000 views.

### 4.3. External Communication

**Figure 6:**
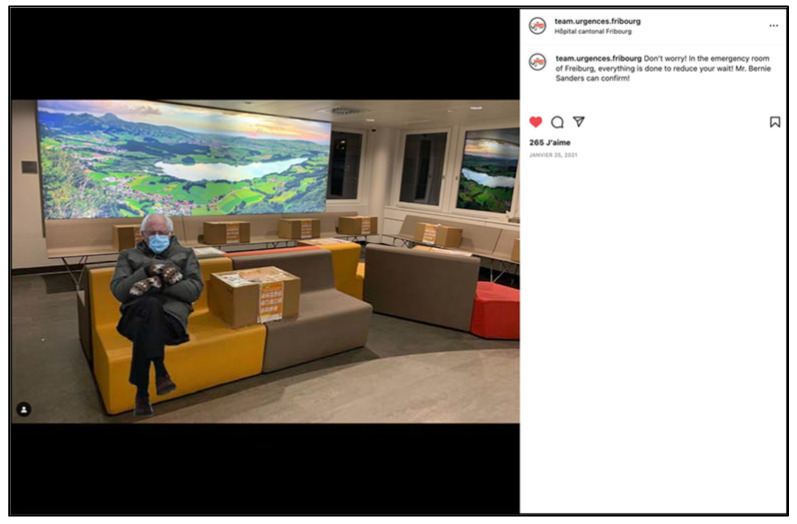
Use of humor to illustrate an information campaign on waiting times at the emergency room in Fribourg (screenshot using a famous image of Bernie Sanders) (screenshot).

In Europe, the waiting period before medical contact is often criticized by emergency room users. In any emergency department, the rule is the same: “when it’s serious, you don’t wait”. For patients with less serious conditions, the order of treatment depends on triage guidelines. The more overloaded the services are, the longer the wait is likely to be, regardless of the dedication of the emergency teams. These guidelines are sometimes ignored by the general public, who nowadays consult emergency rooms day and night to find the answer to their various health problems, often because they have not found the answer to their problem elsewhere. The increase in the flow of incoming patients is not insignificant as the overloading of emergency services and delays before hospitalization are now identified as independent factors of morbidity and mortality [15]. It is also directly correlated with the sense of exhaustion experienced by the teams. Overcrowding is a global issue and Switzerland has not been spared. The slightest malfunction is often over-publicized, sometimes erroneously, with a negative impact on the image of the service and its staff. In its story thread, our Instagram account regularly repeats these messages to its community (Figure 6).

### 4.4. Internal Communication

Disseminating information to teams as large as those of an emergency department working staggered hours is a challenge. All means of expression are suitable (oral communication, departmental meetings, posters, emails, intranet, letters, etc.), but it is often the combination and, above all, the repetition of them that allows for efficient information sharing. With the advent of social networks in recent years, these communication codes seem to have been turned upside down and two generations are now up against each other. The first generation, called Generation Y, was born before 1995 and is made up of “digital adopters” who had to learn digital codes. The second generation born after 1995, known as “Z”, refers to the “digital natives” who have been immersed in these new codes since birth. Younger people, accustomed to the immediacy of networks, seem more sensitive to this type of content in terms of internal communication than to more traditional messages. The transmission of service information via these new channels is interesting to explore. By staging certain non-confidential, filtered information, the management team shares with its team the guidelines of their objectives and results.

### 4.5. Education and Training

**Figure 7:**
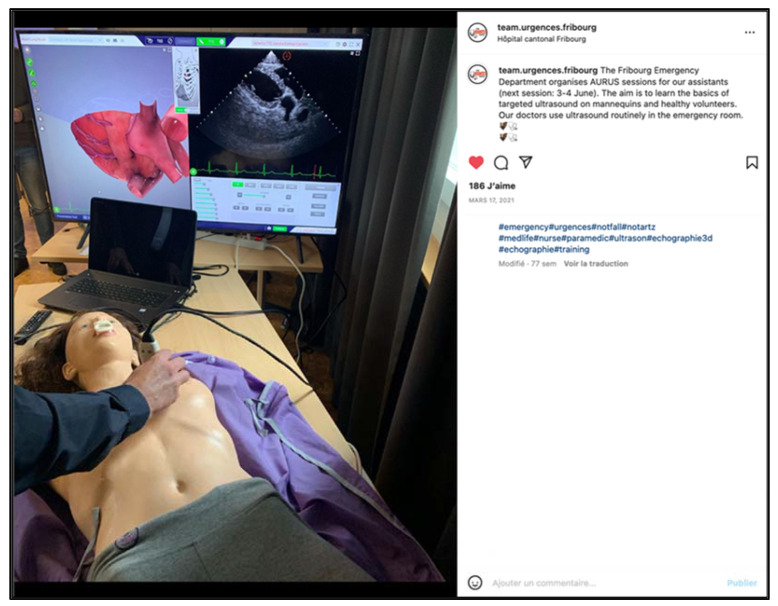
Illustration of the means made available for the student training (here depicting ultrasound) (screenshot).

**Figure 8:**
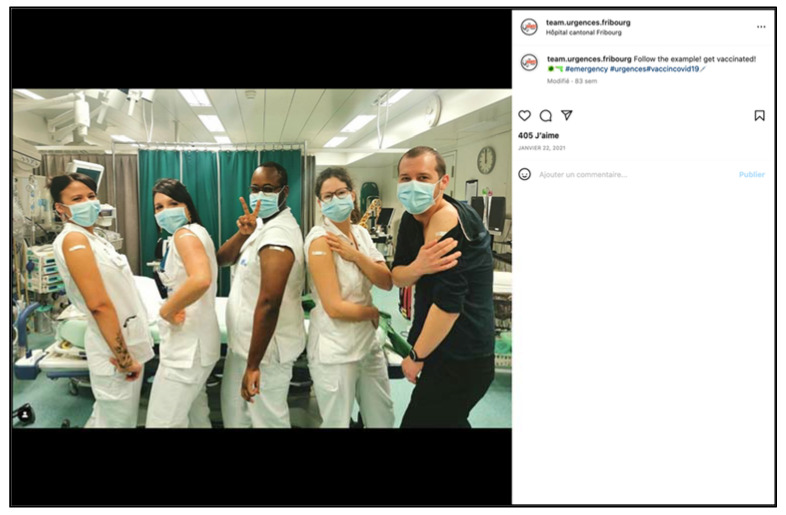
Vaccination campaign during the COVID-19 pandemic (screenshot).

In the field of education and training, social media resources are used frequently by emergency medicine programs and allow a greater dissemination of information and individualized learning [16]. For many years, a large number of accounts have been sharing emergency medicine news, in addition to existing blogs, podcasts and videocasts, with limitless possibilities (quizzes, video tutorials and lives). Our aim was not primarily to develop a teaching and training tool, but the platform is particularly well suited for the transmission of images or short videos for the teaching and prevention or promotion of emergency medicine. In the course of the “stories”, the management team shares or transmits medical news (and guidelines or interesting publications) to its care teams, thus optimizing the screen time of its employees who have become followers. The team also shares its innovations and its commitment to the promotion of new tools such as targeted ultrasound or the implementation of new techniques such as hypnosis (Figure 7). As there are multiple targets (employees and the general public), lessons and prevention messages are given to the general public, but also to the teams (vaccine prevention, life-saving procedures, clinical signs of myocardial infarction, stroke, role of the 144 health center, etc.) (Figure 8).

## 5. Discussion: The Birth of a New Management Concept?

The thread of communication of our emergency department Instragram centers on a “virtual” e-boss described by the account as awful and tyrannical. This figure makes a regular appearance throughout the stories and posts. We know when they are happy or angry, when they work (rarely), when they go on vacation (often), but also when they return (unfortunately) (Figure 6). This character, focused on self-derision du chef, becomes the guideline of the account, but nobody knows who they really are. By portraying its news, successes and difficulties in the form of photographic reports and humorous video metaphors, the account has succeeded in gaining a growing number of loyal followers with whom it interacts, while passing on key messages about the role of an emergency department in the daily care of patients. Beyond care, the account evokes the personal fulfilment of professionals through extra-professional activities or the appeal of the country. The account community quickly brought together team members and hospital staff, local residents and emergency service workers from all over the canton of Fribourg, as well as members of the hospital administration, politicians and some local media (5300 followers in two years compared to 1600 for the hospital’s official account). Interactions with this community and the team members are important, even if it remains difficult to quantify. Each video is viewed more than 4000 times, and some photos have generated more than 700 interactions. As a mark of success, the account was made official by the hospital’s management one year later. The communication unit has approved the tool under the cover of tacit rules of good use (a single account administrator, the head of department, who takes responsibility and observes strict adherence to professional privacy). Acclaimed by the local media and, against all odds, it has become a new management tool for the team.

**Figure 9:**
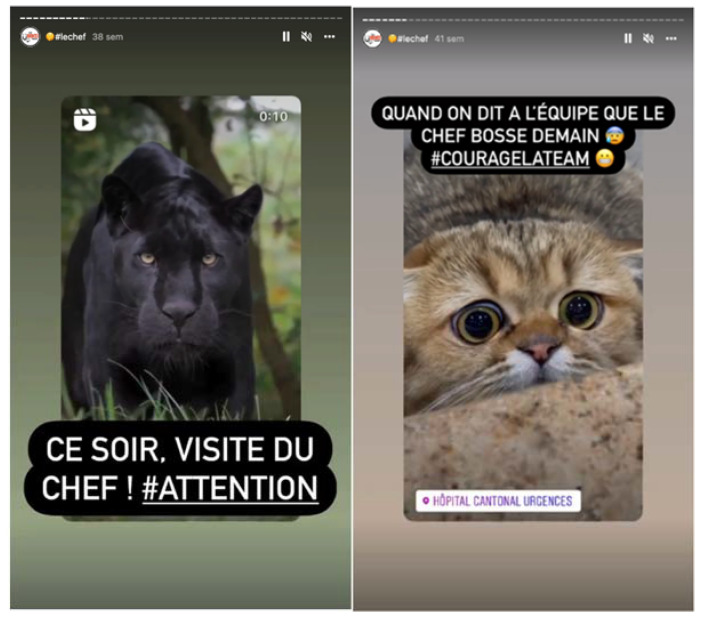
The “e-boss”: a humorous imaginary character, mysterious, sometimes tyrannical, but who pushes his teams towards the best (**Left** screenshot): “When the team knows the boss is working tomorrow #couragetheteam”; (**Right** screenshot): “Tonight, visit of the Chief! #watch out”.

The formidable e-boss, even if tyrannical, encourages their employees and showcases their professionalism (Figure 9). Innovations to cope with the incessant flow of patients are shared. Interactions with followers are increasing and they motivate care teams by sending them messages of encouragement or thanks. Team members congratulate each other by commenting on the publications on the good work accomplished. A new management concept is now being developed and is centered on the positive leadership of this e-boss, with never-ending possibilities. This creates a real corporate culture based on the self-deprecation of a leader that nobody knows. Similar to a hypnosis session, this leader makes direct and indirect suggestions to the team. Humor is a subtle management tool. It relieves stress and brings a little lightness to health professionals who are increasingly under pressure. Today, no one should be unaware of the impact of these tools on the management of health services. On the other hand, it has been shown that new competencies about the use of social media are important for health professionals, including knowledge, skills and attitudes such as e-professionalism and the development of strategies for effective communication on social media (how to get others to engage with your content) [17]. Finding good strategies related to the practice of emergency medicine in an evolving digital world is mandatory. Even if our first results seem encouraging (adhesion of the team, positive feedback from the population and the local media, application of doctors through this network...), the interpretation remains subjective. To our knowledge, there is no description of the use of social networks in team management. It is therefore difficult to support or compare the concept with existing literature. It will be necessary to ensure that its results are reproducible by other teams and, importantly, study the results over time. Managers should, however, be inspired by companies that routinely use social networks to promote their product or brand in order to potentiate their leadership. The E-Boss could well be the ideal leader and a real help in building a team. This new management concept’s place must be understood if it is to be successfully integrated into existing tools. Its impact on management should be demonstrated in order to move beyond the concept.

## 6. Conclusions

The inherent risks of allowing a team of health professionals to communicate about their daily lives through a social media platform are certain: non-respect of medical confidentiality; ethics; missteps or slip-ups; and potential damage to the institution’s image. The medical literature also tends to emphasize this problem rather than questioning the positive nature of these new tools. When the hospital institution adopts a relationship of trust with its employees in their communication strategy under the cover of tacit agreements of good use to maintain proper standards of ethical and professional conduct, the results can quickly exceed expectations and the tool can become a real daily management tool. Our experience is very positive, and the account and its e-boss continue to encourage care teams, promote the profession of emergency service workers, and facilitate recruitment. In a world where there is a growing shortage of caregivers, an innovative approach is mandatory to attract staff and social media can help. When the social media manages to unite a significant number of followers around a virtual leader, including team members, members of the hospital’s management, including some media or politicians and people from your local area, the gamble has probably paid off. The e-boss exercises positive leadership and becomes an “influencer” and the management is much more effective. The management of hospitals and emergency departments is changing, and social media certainly has a role to play in their future evolution. In conclusion, we are reporting the first feedback on the use of social networks as tool for the management of an emergency department, but this feedback proposed as a new concept should be subject to further study or evaluation.

## Data Availability

Not applicable.
